# Single-Walled Carbon Nanohorns as Promising Nanotube-Derived Delivery Systems to Treat Cancer

**DOI:** 10.3390/pharmaceutics12090850

**Published:** 2020-09-07

**Authors:** Alazne Moreno-Lanceta, Mireia Medrano-Bosch, Pedro Melgar-Lesmes

**Affiliations:** 1 Department of Biomedicine, School of Medicine, University of Barcelona, 08036 Barcelona, Spain; amorenol@clinic.cat (A.M.-L.); mireiamb_7@hotmail.com (M.M.-B.); 2 Biochemistry and Molecular Genetics Service, Hospital Clínic Universitari, IDIBAPS, CIBERehd, 08036 Barcelona, Spain; 3 Institute for Medical Engineering and Science, Massachusetts Institute of Technology, Cambridge, MA 02139, USA

**Keywords:** single-walled carbon nanohorns, cancer, nanotube, carbon, therapy

## Abstract

Cancer has become one of the most prevalent diseases worldwide, with increasing incidence in recent years. Current pharmacological strategies are not tissue-specific therapies, which hampers their efficacy and results in toxicity in healthy organs. Carbon-based nanomaterials have emerged as promising nanoplatforms for the development of targeted delivery systems to treat diseased cells. Single-walled carbon nanohorns (SWCNH) are graphene-based horn-shaped nanostructure aggregates with a multitude of versatile features to be considered as suitable nanosystems for targeted drug delivery. They can be easily synthetized and functionalized to acquire the desired physicochemical characteristics, and no toxicological effects have been reported in vivo followed by their administration. This review focuses on the use of SWCNH as drug delivery systems for cancer therapy. Their main applications include their capacity to act as anticancer agents, their use as drug delivery systems for chemotherapeutics, photothermal and photodynamic therapy, gene therapy, and immunosensing. The structure, synthesis, and covalent and non-covalent functionalization of these nanoparticles is also discussed. Although SWCNH are in early preclinical research yet, these nanotube-derived nanostructures demonstrate an interesting versatility pointing them out as promising forthcoming drug delivery systems to target and treat cancer cells.

## 1. Introduction

Cancer is characterized by defects in the regulatory mechanisms that govern the growth and homeostasis in normal cells, causing sustained and abnormal proliferation [1]. It is the second leading cause of death worldwide after cardiovascular disease and its incidence is increasing [2]. Surgery and radiotherapy are the primary treatment choice in localized solid tumors [3], whereas chemotherapy is widely used in systemic malignances [4]. Biotechnological therapies, such as immunotherapy and oncolytic viruses, have also been developed in the last decades to combat this disease [5]. However, these therapies show the same disadvantage than pharmacological strategies, as they are not specifically targeted to the tumor tissue. This entails different problems derived from the high toxicity in healthy tissues and highlights the importance of the development of tumor-targeted strategies. This is one of the main reasons that explains the exponential growth in the number of new designs of nanomedicine delivery systems in the last decades for the treatment of cancer [6,7].

Multiple targeted drug delivery systems (DDS), such as liposomes, micelles, polymers, inorganic carriers, hydrogels, or macromolecular scaffolds have been developed so far [8]. They can be constructed as tissue-specific therapeutic systems, thus reducing the systemic toxicity. They confer the possibility of targeting other cell types associated to the tumor microenvironment, such as cancer-associated fibroblasts (CAF) [9] or tumor-associated macrophages (TAM) [10]. DDS at the nanoscale benefit from the enhanced permeability and retention (EPR) effect, which consists of the intratumoral accumulation of particles due to the increased number of blood vessels formed during the angiogenesis process driven by tumor-released factors [8,11,12].

Several types of materials have been employed and tested for the development of nanocarriers in preclinical models of cancer. Among inorganic materials, the most frequently used are gold, iron, or carbon-based materials [13]. Single-walled carbon nanohorns (SWCNH) are carbon-based materials composed of single graphene sheets forming conical and horn-shaped tips. During their synthesis, they tend to form aggregates of clustered SWCNH [14]. The biomedical applications of SWCNH have increased in the recent years, as well as other carbon-based materials such as carbon nanotubes or fullerene. SWCNH do not require metal catalysts for their synthesis and can be produced in industrial quantities. SWCNH display own unique characteristics due to their specific conical morphology and surface chemistry (this latter shared by other graphene nanoparticles) that enable drug loading and targeted release. Conical morphology with a wide apex angle and distorted tubule drastically influences the electronic and magnetic properties and reactivity, showing at least one unpaired electron spin. Moreover, conical shape can help to entrap drugs or other substances into the structure and the tip can be chemically functionalized by the attachment of targeting moieties. These properties highlight SWCNH as an attractive alternative to carbon nanotubes and graphene oxide in a wide range of biomedical applications. Namely, SWCNH have the potential to become one of the most useful carbon nanotube-based materials for the design of targeted therapeutic strategies to combat cancer [15]. This review summarizes the SWCNH structure and types of functionalization, as well as the possible applications in the fields of biomedicine and drug therapy, emphasizing its use in cancer therapy.

## 2. Single-Walled Carbon Nanohorns (SWCNH)

### 2.1. Definition and Structure of SWCNH

**Figure 1:**
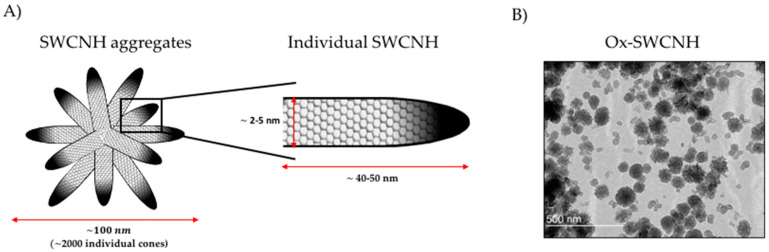
(**A**) schematic illustration of the structure of SWCNH; (**B**) representative image of carboxylated nanostructures obtained by Transmission Electron Microscopy (TEM). TEM image reprinted with permission from [21] (Melgar-Lesmes, P.; et al. Graphene-Dendrimer Nanostars for Targeted Macrophage Overexpression of Metalloproteinase 9 and Hepatic Fibrosis Precision Therapy. Nano Lett. 2018, 18, 5839–5845, doi:10.1021/acs.nanolett.8b02498), Copyright (2018) American Chemical Society.

SWCNHs are carbon conical horn-shaped nanostructures constructed from sp2 carbon sheets (graphene sheets), discovered by Iijima et al. in 1999 [16]. Individual conical structures are typically 2–5 nm in diameter and 40–50 nm in length. During their synthesis, they tend to form about 2000 cone aggregates of approximately 100 nm diameter [14,17] (Figure 1). They have a number of advantages over the use of carbon nanotubes—extensively utilized carbon-based structures for drug delivery [18,19] such as the absence of potentially toxic metals as catalysts during the synthesis, unnecessary additional treatment with strong acids that can damage the carbon structure, and the capacity for high yield mass production at room temperature [14,20].

### 2.2. Synthesis

Since its discovery, multiple synthesis approaches have been developed. All methods are based on applying energy to disassemble and reorganize carbon structures, which are usually graphite rods [14]. The different working parameters modulated during the synthesis, such as voltage, intensity, pressure and temperature, can result in different SWCNH structures with different morphology, size or purity [17,22]. Three different type of nanoaggregates have been described in particular: dahlia-like, bud-like, and seed-like SWCNH [20].

CO_2_ laser ablation was the first synthetic method used for the discovery and development of SWCNH [16]. This high-yield synthesis procedure modifies graphite targets, without any metal catalyst, producing up to 1 kg SWCNHs per day with 95% purity [23]. Since then, it has been one of the most exploited strategies for its production. Arc-discharge has also been proposed for their relatively low-cost synthesis. An electrical discharge is emitted between two electrodes subjected to a difference in potential and placed in a gaseous atmosphere. The electric arc may be formed under air [24,25], CO or CO_2_ [26] atmospheric pressure. This technique offers the possibility to obtain purity values higher than 90%. Arc-discharge can also be performed between two graphite electrodes immersed in liquid nitrogen, resulting in a very economical alternative to the classical method [27]. Finally, the use of reactors where graphite rings are heated by the induction of high frequency eddy currents has also been proven as a powerful and useful strategy for large-scale production of SWCNH [28]. In general terms, CO_2_ laser ablation and arc-discharge have been the most used methods since the discovery of SWCNH for its development.

### 2.3. Properties of SWCNH

SWCNHs have several interesting features that have been exploited for a multitude of applications. SWCNH display a porous structure with a very high adsorption capacity. Controlled oxidation treatments can produce nano windows within SWCNH tips and lateral walls. For this reason, they have been proposed for gas storage and gas sensing applications, such as N_2_ [29,30] and H_2_ [31]. These structure windows due to oxidation can also be formed as a previous step for chemical functionalization, and then include various functional groups for other applications [32]. The large and tunable surface area of SWCNH, together with the great capacity for heat and electrons transport, also make them interesting for both conversion and energy storage [33] applications. They have also been employed in the field of electronics due to their cone structure and electric features. Several studies have revealed that SWCNH present structural defects in the tips of individual nanohorns, with a series of heptagons instead of pentagons that form the two-dimensional graphene sheets. These defects are essential to exhibit their special electronic and magnetic characteristics [34]. Hence, they have been used for the development of electrodes and supercapacitors [35], fuel cells [36], and catalyst supports [37]. Versatile surface chemical functionalization of SWCNH has also been exploited to develop new biomedical and pharmacological strategies in recent years.

### 2.4. Non-Covalent and Covalent Functionalization of SWCNH

Physicochemical properties of synthesized SWCNHs can be modulated by different chemical functionalizations (in the walls of the tips) for biomedical applications. Diverse functionalized nanoaggregates may be developed and employed for a multitude of biomedical and pharmacotherapeutic approaches. Functionalization has been proposed as a strategy to regulate SWCNH stability and solubility in different solvents, since they present an extremely hydrophobic surface area [14,17]. Overall, the types of functionalization can be classified in two broad categories: covalent and non-covalent modifications.

Non-covalent functionalization refers to electrostatic interactions or those in which non-covalent π–π stacking occurs between SWCNH side walls with aromatic organic rings. At this point, π-conjugated system is maintained without breaking the nanostructure, which is especially important for their use in electronics. Interestingly, non-covalent interactions between SWCNH and aromatic rings may result in molecule stabilization, as occurs in hybrids between porphyrin molecules and SWCNH. Tetra cationic water-soluble porphyrin had been attached to the nanohorn skeleton by π–π stacking interactions, resulting in an attraction between aromatic rings. Indeed, a stable solution in water could be obtained without altering the unique electronic network of the nanoaggregate [38]. Positively charged pyrene units have also been described as a way to solubilize SWCNH in water for electronic applications [39].

The attachment of polymers such as amphiphilic lipid-based poly (ethylene glycol) has also been suggested to produce water-stable SWCNH through non-covalent functionalization [40]. Similarly, another recent study has proposed to modify SWCNHs with poly (maleic anhydride-*alt*-1-octadecene) and methoxypolyethylene glycol-b-poly(d,l-lactide), to sequentially load different drugs for combined chemo-photothermic therapy [41]. Other authors have used a block polyelectrolyte wrapping strategy for the development of a soluble nanohybrid, stable for several months. In this case, a hydrophobic polystyrene block has been attached to SWCNH by hydrophobic interactions and, in turn, it has been stabilized by amphiphilic poly [sodium (2-sulfamate-3-carboxylate) isoprene-b-styrene] block polyelectrolyte [42]. More recently, binding SWCNH to photochromic molecules has been studied for molecular electronics, in which a non-covalent interaction between the nanostructure and 4-[4-dodecyloxycarbonyl-2-pentadecylphenylazo] benzoic acid has been established by previous oxidation by the treatment with H_2_O_2_ at 60 °C for 5 h. Then, oxidized SWCNH (ox-SWCNH) were attached to the photochromic molecule by hydrogen bonds forming a non-covalent hybrid [43].

SWCNH can also be functionalized through the covalent union of organic molecules, conforming more stable units, modifying nanoparticle walls and/or forming tuned open conical ends. Oxidation processes are also commonly used to increase solubility, both in water and in organic solvents, and reactivity with other molecules, due to the formation of carboxyl groups (R-COOH) among others. For example, light-assisted oxidation with H_2_O_2_ leads to carboxyl group addition in SWCNH, promoting the opening of holes in their walls. For this, 400 mg of as-grown SWCNH were treated with H_2_O_2_ placed on a hot plate at 100 °C and irradiated with a xenon lamp at an intensity of 3W [44]. Pretreatment with diluted HNO_3_ under microwave irradiation resulted in oxidation, so their dispersion in water was facilitated [45]. Furthermore, thermal treatment in an O_2_ atmosphere opened and broke SWCNH structure so that its surface area increased to introduce palladium nanoparticles [46,47].

**Figure 2:**
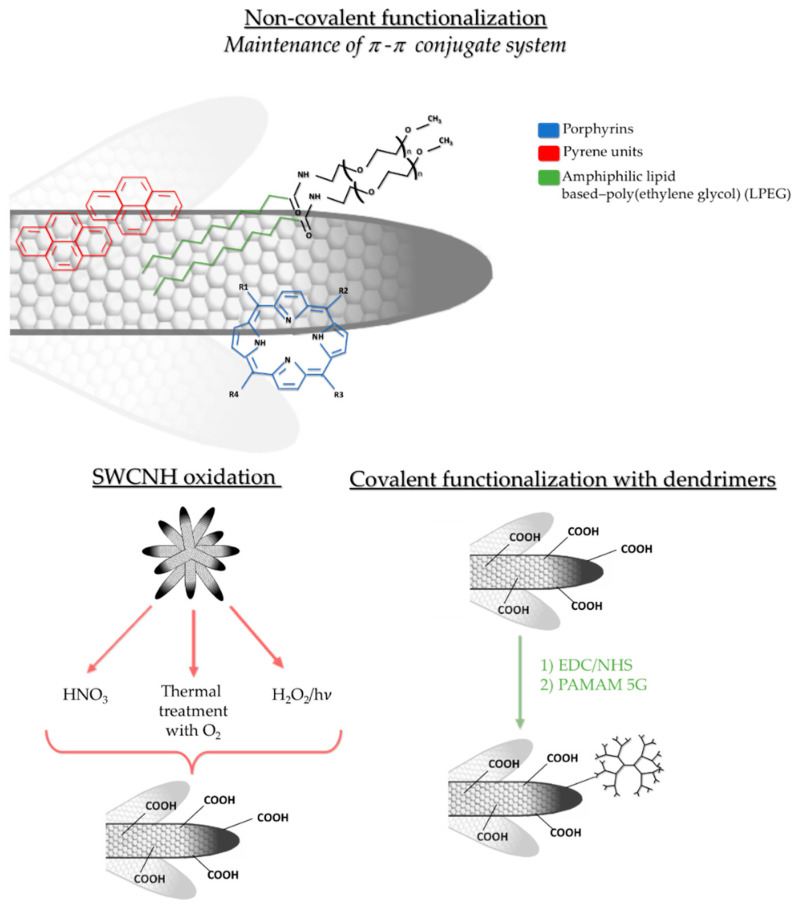
Non-covalent and covalent functionalization of SWCNH. Different types of non-covalent functionalization with porphyrins, pyrene units and amphiphilic lipid-based poly(ethylene glycol). Schematic oxidation for carboxylic group formation and covalent functionalization with fifth generation poly(amidoamine) (PAMAM) dendrimer.

Other molecules can be attached to ox-SWCNH, such as dendrimers or quantum dots, in order to design drug delivery strategies or nano-theranostic nanoparticles. For example, ox-SWCNH have been simultaneously modified to perform both fluorescence imaging and cisplatin delivery for bladder cancer lesions [48]. After HNO_3_ oxidation, carbodiimide reaction was carried out with 1-Ethyl-3-(3-dimethylaminopropyl) carbodiimide (EDC) and *N*-Hydroxysulfosuccinimide sodium salt (sulfo-NHS). Finally, the nanoaggregate was decorated with cysteamine hydrochloride (AET). Cisplatin acted as a therapeutic agent and SWCNH-AET-hybridized quantum dots served for fluorescent diagnostics [48]. The same EDC/NHS reaction has been used to attach generation 5 poly(amidoamine) (PAMAM 5G) to target, tag, and treat inflammatory macrophages with an expression plasmid in liver fibrosis [21]. Other types of nanostructures can be deposited within SWCNH. For example, Pt nanoparticles have been introduced into the structure after breaking the nanohorn walls with concentrated HNO_3_ [49]. Indeed, constructs of SWCNH and Pt attached to PAMAM dendrimers have demonstrated that they can be an interesting strategy to build electrochemical immunosensors for the detection of procalcitonin and the rapid diagnosis of sepsis [50]. The common covalent and non-covalent functionalizations are represented in Figure 2.

## 3. Use of SWCNH in Biomedicine: Drug Delivery and Gene Therapy

Targeted controlled drug release is necessary to improve both therapeutic efficacy and tolerance when treating different human diseases [20]. Many types of nanoparticles have been reported to be potential candidates as drug carriers but low production yields, instability in the physiological environment or low ability to efficiently adsorb drugs have hampered their widespread use as drug carriers so far [51]. Among them, SWCNH aggregates have been suggested as potentially appropriate drug carriers because of their stability, inertness, and large surface area [20,51]. Other applications in the medical field include magnetic resonance analysis and photodynamic therapy [52].

### 3.1. Biocompatibility and Toxicity of SWCNH

One of the most intriguing features of SWCNHs is that they have extensive surface area and multitudes of horn interstices, which enable large amounts of adsorbed molecules [53] and provides suitable sites to incorporate and protect drugs [51]. Moreover, the surface area can be further enlarged by oxidation, from 400 m^2^/g of carbon nanohorns to 1400 m^2^/g in oxidized carbon nanohorns. This causes the aperture of holes on their walls, allowing the infiltration of small molecules in their inner spaces and allowing the slow and controlled release of the infiltrated molecule [20]. Oxidation, aside from introducing oxygen functional groups that can be used to functionalize these DDS, facilitates SWCNH excretion [20,53]. The straightforward and cost-benefit process to obtain SWCNHs in large amount with a high purity [54] also makes SWCNHs an attractive material for medical use [53]. Moreover, SWCNH, which does not require metal catalysts for synthesis, demonstrates excellent biocompatibility properties [52,54]. SWCNHs have not shown any measurable toxicity in tests performed in animals evaluating the carcinogenic potential, the dermal and ocular reactions, the peroral or intragastric effects [55], and pulmonary toxicity [56]. Intravenously injected functionalized nanohorns via mouse tail vein did not show harmful toxicity [21,57]. The results of all these tests have confirmed that SWCNHs should have a negligible toxicity on living beings [55], but further studies are required. The dose, the period of mouse monitorization, and the evaluated parameters for each administration route and different toxicity tests are described in Table 1.

**Figure 3:**
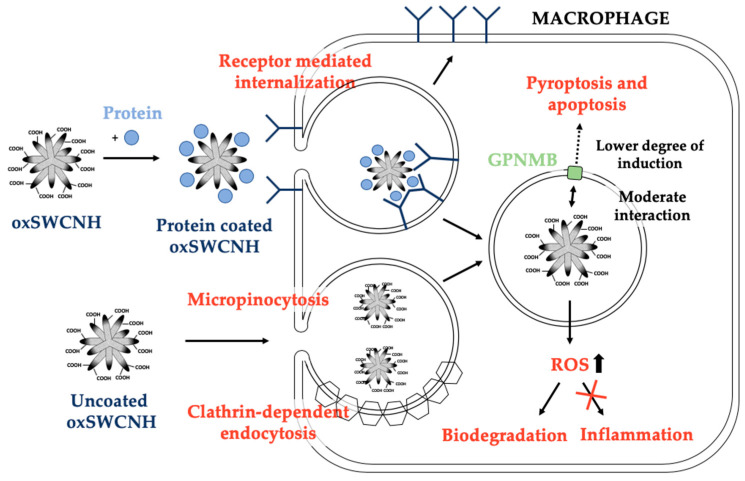
SWCNH uptake processes in macrophages. SWCNH are mainly engulfed by activated macrophages via direct trans-membrane uptake or receptor-mediated processes. Protein-coated SWCNHs follow a receptor-mediated internalization and SWCNHs without coating biomolecules can be internalized by micropinocytosis or clathrin-dependent endocytosis. Macrophages, after the uptake of SWCNHs, generate reactive oxygen species (ROS) to achieve the degradation of the nanomaterials without triggering any inflammatory pathway during this process. On the other hand, SWCNHs have a moderate interaction with glycoprotein nonmetastatic melanoma protein B (GPNMB) inducing a lower degree of cascade actions to trigger pyroptosis and apoptosis in macrophages.

After administration, SWCNHs can cross the first biological barrier of polar epithelial cells by transcytosis due to their strong bioadhesion properties allowing its spread throughout the body [58]. Although with low cytotoxicity, evidence has shown that these compounds are trapped by the liver, lung, or spleen after administration to animals [20], especially in mononuclear phagocytic system cells, such as macrophages [59]. Oxidation of SWCNH has demonstrated to be an excellent solution to enhance biocompatibility and improve excretion [44]. Physiological oxidation by peroxidase enzyme-based processes additionally contributes to the biodegradation of carbon nanotubes and graphene [11,12]. Macrophages also play an important role on metabolism and excretion of SWCNHs [59]. Macrophages incorporate SWCNHs by endocytosis [60] and generate reactive oxygen species (ROS) to achieve the degradation of these nanomaterials, but without triggering any inflammatory pathway during this process. Actually, inflammation markers such as pro-inflammatory cytokines interleukin 6 (IL-6) and tumor necrosis factor (TNF) are not induced by SWCHN [59]. It has also been found that SWCNHs display a weaker interaction with glycoprotein nonmetastatic melanoma protein B (an important protein in nanotoxicity initiation) compared to carbon nanotubes. Hence, SWCNH induces a lower degree of cascade actions to trigger pyroptosis and apoptosis in macrophages [60]. SWCNH may behave as single-walled carbon nanotubes (SWCNT) in terms of macrophage uptake mechanistic [61]. SWCNH and SWCNT uptake is the result of both membrane uptake and receptor-mediated processes. SWCNTs are often associated with serum proteins (such as albumin and globulin) that can be recognized by the numerous receptors expressed on the surface of macrophages and subsequently internalized [61]. Protein-free SWCNTs are mainly internalized by micropinocytosis [62]. However, alternative pathways such as clathrin-dependent endocytosis have been reported to be important for internalizing shorter species of SWCNTs [62] (Figure 3).

### 3.2. Biomedical Applications of SWCNH

Different studies have demonstrated the potential of SWCNH as DDS. Murakami et al. investigated the ability of SWCNHs to bind and release the anti-inflammatory glucocorticoid dexamethasone. First of all, they compared the binding ability of SWCNHs and oxidized SWCNHs (oxSWCNHs) demonstrating that the adsorption capacity of oxSWCNHs was approximately six times higher to achieve the desired anti-inflammatory effects [53]. Hence, oxidized SWCNH exhibited sustained release of biologically active dexamethasone in mammalian cells without significant side effects. Another study has reported that prednisolone (PSL), another anti-inflammatory glucocorticoid drug, could be adsorbed on oxSWCNHs and gradually released from them according to the change in the local concentration of PSL [54]. Then, to prove the in vivo biological effect of PSL, these ox-SWNHs containing PSL were injected in tarsal joints of rats with collage-induced arthritis. Retardation of arthritis progression compared with controls was observed due to the anti-inflammatory effect of PSL-oxSWNHs. Moreover, they proposed that PSL-oxSWNHs might have an immunoregulatory function because they accumulated into inflammatory macrophages [54].

OxSWCNH can also incorporate antibiotics as vancomycin hydrochloride and release it in a slow and stable manner for an extended period. Phospholipid–polyethylene glycol (PL-PEG) and amination have been used to modify oxSWCNH to improve dispersion in aqueous systems [20,51]. The properties confined by PEG included thermal stability, resistance to proteolysis, increased water solubility, decreased antigenicity and immunogenicity, and a slower clearance from plasma.

SWCNHs have also shown a catalytic activity when functionalized with other chemical groups. For example, Zhu et al. described that carboxylic-group-functionalized SWCNHs (SWCNHs-COOH) possess a peroxidase-like activity. The SWCNHs-COOH can catalyze the oxidation of the peroxidase substrate 3,3′,5,5′-tetramethyl benzidine (TMB) in the presence of H_2_O_2_ to generate oxidized 3,3′,5,5′-tetramethyl benzidine (TMB), which exhibits a blue color change in aqueous solutions. Combining the SWCNH-COOH catalytic reaction and the generation of H_2_O_2_ by the enzymatic oxidation of glucose with glucose oxidase, they developed a colorimetric method for glucose detection. This method exhibited a highly selective response to glucose detection and also potential applications in clinical diagnosis [63].

The application of SWCNH as potent laser therapeutic agents for the highly selective elimination of microorganisms has also been reported by Miyako et al. who designed a complex formed by SWCNH and a molecular recognition element to selectively target the microbial surfaces, and PEG chains to increase the water dispersibility of the complexes. The complexes bound the microbial cell and then they were destroyed by triggering an exothermic reaction to near-infrared (NIR) laser irradiation [64]. SWCNH converts NIR photon energy into thermal energy to achieve the elimination of microorganisms. In fact, functionalized SWCNH nanomaterials have also demonstrated photo-exothermic properties for elimination of harmful viruses [65].

SWCNH applications for biosensing have also been investigated in recent years, for example, to fabricate a glucose biosensor [66] constructed by encapsulating glucose oxidase in a Nafion–SWCNHs composite film. SWCNH have also been used for the simultaneous electrochemical determination of uric acid, dopamine, and ascorbic acid [67]. They have also been employed for guiding bone regeneration [68] and to enhance osteoblast functions and cell–substrate interactions, prompting soft-tissue reconstruction and replacement [69].

**Figure 4:**
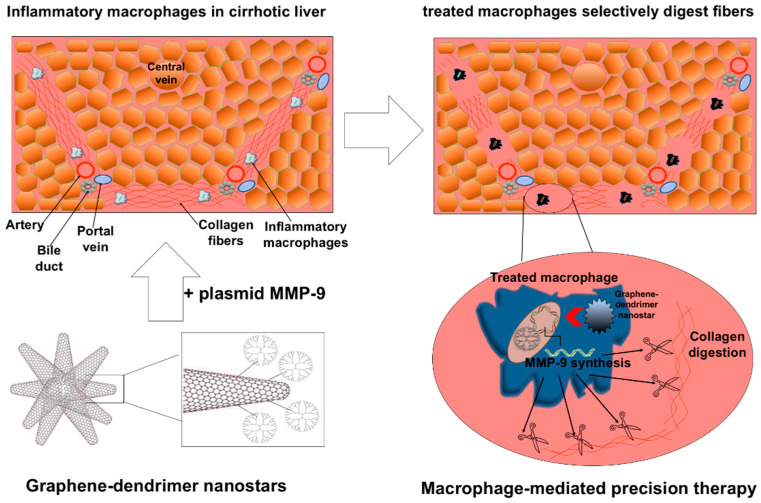
Targeted gene therapy with Graphene-dendrimer nanostars formed by SWCNH. Plasmid metalloproteinase 9 (MMP-9) is selectively delivered into inflammatory macrophages in cirrhotic liver by dendrimer-graphene nanostars allowing the synthesis and excretion of a functional MMP-9 to digest adjacent collagen fibers. Reprinted with permission from [21] (Melgar-Lesmes, P.; et al. Graphene-Dendrimer Nanostars for Targeted Macrophage Overexpression of Metalloproteinase 9 and Hepatic Fibrosis Precision Therapy. Nano Lett. 2018, 18, 5839–5845, doi:10.1021/acs.nanolett.8b02498), Copyright (2018) American Chemical Society.

Another biomedical use of graphene-based nanomaterials is targeted gene therapy. As described above, PAMAM dendrimers have also been attached to SWCNH. They have the ability to interact with genetic material and successfully deliver the genetic cargo into the cytoplasm, leading to variations in the cell gene expression [70,71]. SWCNH have been used to deliver small interfering RNA (siRNA) to achieve therapeutic silencing and downregulation of specific gene expression. Al-Jamal et al. demonstrated that carbon nanotubes, functionalized by introducing an ammonium group, could bind siRNA and induce in vivo gene silencing of Caspase-3 leading to neuroprotection and significant functional improvement before and after ischemic insult [72]. In another study, SWCNH-PAMAM dendrimers attached to siRNA were used to stimulate cell death through the knockdown of cofilin-1 via caspase-3 activation [73]. SWCNH can also be used to increase and upregulate gene expression. Graphene nanostars consisting of SWCNH linked to PAMAM dendrimers allowed plasmid delivery for selective gene expression of the collagenase metalloproteinase 9 into inflammatory macrophages in cirrhotic livers [21]. Metalloproteinase 9 overexpression promoted the phenotypical transformation from inflammatory to pro-regenerative macrophages (Figure 4). This targeted gene therapy reduced selectively and locally the presence of collagen fibers and decreased hepatic injury [21]. These evidences has shown that functionalized SWCNH could be used on the therapy of diseases associated with fibrosis and inflammatory macrophage accumulation.

## 4. SWCNH in Cancer Therapy

Current use of SWCNH for cancer therapy can be classified as follows: SWCHN as antitumoral nanoparticles; nanosystems for targeted use of chemicals or drugs against cancer cells; photothermal and photodynamic treatment; gene therapy (increase or interference in gene expression); and immunosensing.

### 4.1. SWCNH as Anticancer Nanoparticles

SWCNH have been reported to display intrinsic antitumoral biological activity in some cancer cell line models. In contrast, no toxic effects have been observed in healthy human umbilical vein endothelial cells using concentrations from 5 to 500 µg/mL [21]. In tumorigenesis, inhibition of apoptosis is one of the hallmarks for cancer development [1]. SWCNH have demonstrated to be apoptosis inducers in HepG2 hepatoma cancer cells. In this case, the authors showed that SWCNH aggregates were able to enter the mitochondrial matrix and change the distribution of protons and ions on both sides of the internal membrane. This would lead to the depolarization of the membrane causing apoptosis [74]. Another study has also reported that small amounts of unmodified SWCNH could suppress cell proliferation, affect cell cycle, and promote apoptosis in a dose-dependent manner in this same hepatoma cell line [75]. A study where HepG2 cells were implanted in a nude mice animal model and treated with SWCHN demonstrated an increased endoplasmic reticulum-related stress, although not at the tumoral level [76]. Therefore, SWCNH could lead to endoplasmic reticulum stress or trigger intrinsic mitochondrial-dependent apoptosis in the tumor cells, causing the loss of their tumorigenic capacity. As for other types of tumors, the conjunctival cancer cell line CRMM1 has also been studied. Here, SWCNH were able to reduce cancer cell proliferation in a dose-dependent manner in addition to an increase in apoptosis and an inhibition of energy metabolism [77].

### 4.2. SWCNH as Delivery Systems for Chemotherapeutic Drugs

Chemotherapy is usually the first-line treatment in cancer. However, dose must be carefully controlled and reduced to avoid devastator side effects in healthy organs, thus limiting anticancer efficacy. SWCNH are an alternative to design DDS for chemotherapeutics (Table 2). Drugs can be released more slowly using SWCNH in the damaged tissue as nanoparticles tend to be retained in the tumor due to the EPR effect [11,78]. This beneficious pharmacokinetic effect could minimize the administered dose of the desired drug, and possibly reduce any associated toxicity [78].

#### 4.2.1. Chemotherapeutic Binding to SWCNH for Cancer Therapy

Cisplatin is a widely used chemotherapeutic drug for the treatment of various human cancers. Acting as an alkylating agent, it interferes with DNA repair systems and causes damage to the molecular structure. Unfortunately, it has many side effects such as hemorrhages, allergic reactions and gastrointestinal and kidney associated problems, among others [48,88]. Ajima et al. have developed a nanoprecipitation method where cisplatin and oxSWCNH were attached in an aqueous dispersion. The agent entered through the open holes when mixed with oxSWCNH helped by sonication. They were precipitated together using distilled water as a solvent. Cisplatin@SWCNH exhibited greater antitumor efficacy than free drug both in vitro (in lung cancer cells) [81] and in vivo in Balb/c nu/nu mice with subcutaneous tumor formed from NCL-H460 human lung cancer cells. Histological examination reported that the nanoconstruct remained in the tumor area for 25 days [78]. Thus, the binding of SWCNH to a chemotherapeutic agent can increase the half-life of the chemical in the tumor tissue, providing obvious benefits in efficacy and activity. The structure of the agent was maintained inside the nanoparticle holes and there was a slow release in aqueous solutions, both in PBS and in culture medium [78,81].

Another chemotherapeutic drug that has been considered as a candidate to incorporate to SWCNH is doxorubicin (DOX). This is a first-line option for some types of cancers, but with considerable side effects, such as lethal cardiotoxicity or myelosuppression [89]. Muramaki et al. have functionalized oxSWCNH with PEG bound to DOX (PEG-DOX), resulting in major solubility in water [84]. PEG-DOX-SWCNH was then intratumorally administered to mice with tumors from human non-small lung cancer cells. A delay in cancer cell growth was observed, and a higher intratumoral drug retention. Only 0.57% PEG-DOX remained after 21 days in the tumor, but, interestingly, PEG-DOX-SWCNH offered a much higher tumor retention (61%) after the same period [85].

#### 4.2.2. Targeted Chemotherapy Using SWCNH

**Figure 5:**
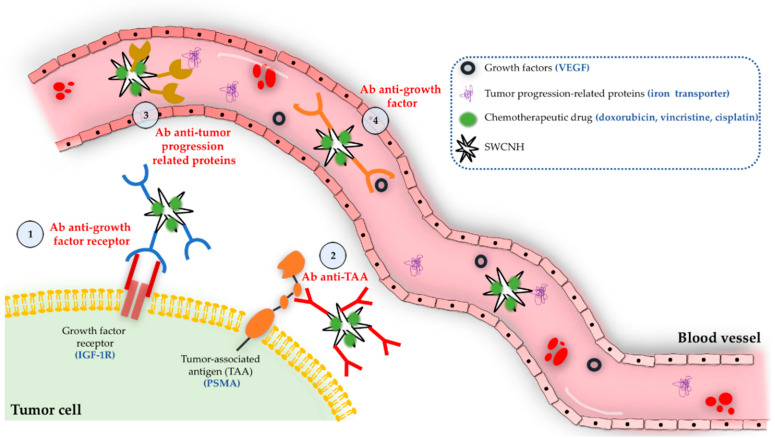
Four different strategies to perform tumor-specific targeting using SWCNH containing chemotherapeutic agents. Targeting can be carried out considering reliable growth factors for tumorigenesis, growth factor receptors, tumor-associated antigens (TAA), or proteins that are essential for tumor initiation and continuation. The image also illustrates various examples of possible targeting molecules.

Selective drug targeting has been carried out by attaching tumor-related molecules to the surface of SWCNH. Different strategies to perform specific tumor targeting are illustrated in Figure 5. For example, vascular endothelial growth factor (VEGF) has a very important role in neovascularization and angiogenesis in the carcinogenic process [90]. Yang et al. have reported a conjugate between DOX and SWCNH that was bound to an anti-VEGF monoclonal antibody. The designed therapy was especially selective for the areas where angiogenesis was more present. Similar results were found in H22 cancer cells subcutaneously injected to ICR mice [82]. Insulin-like growth factor-1 receptor (IGF-1R) also plays a very important role in tumorigenesis since it is involved in stimulating tumor growth and prevents apoptosis. Furthermore, it is overexpressed in many types of malignances such as breast, prostate, liver and glioblastoma cancers [91,92,93,94]. Therefore, IGF-1R could be potentially a cancer targeting molecule. Li et al. have developed vincristine-conjugated SWCNHs attached to an antibody against IGF-1R. The administration of this conjugate to ICR mice subcutaneously implanted with hepatoma cells resulted much more effective than free drug alone and showed less toxicity with higher effective dose delivered into the tumor [87].

Specific tumor targeting can also be designed using tumor associated antigens. For example, prostate-specific membrane antigen (PSMA) is a glycoprotein that remains in cellular cytosol in normal prostate cells but becomes a membrane protein in prostate carcinomas [95]. Lucio et al. have devised a strategy consisting of cisplatin-conjugated SWCNHs attached to an antibody against PSMA-positive prostate cancer cells to promote a selective binding and uptake of the conjugate by positive PSMA cells without reporting any effect on negative PSMA cells [79]. In addition, other types of molecules or enzymes may also be studied for tumor targeting. Transferrin is an iron transporter protein and the expression of its receptor (transferrin receptor 1) has shown to be increased up to 10–100 times in various types of cancer, including breast and lung [96]. Thus, SWCNH conjugated to methotrexate and covalently grafted to transferrin resulted in a much higher adhesion of these conjugates to MAD-MB-321 breast cancer and HepG2 hepatoma cells in comparison to free drug, thereby requiring less chemotherapeutic dose to achieve a similar efficacy [86].

### 4.3. SWCNH in Photothermal and Photodynamic Cancer Therapy

**Figure 6:**
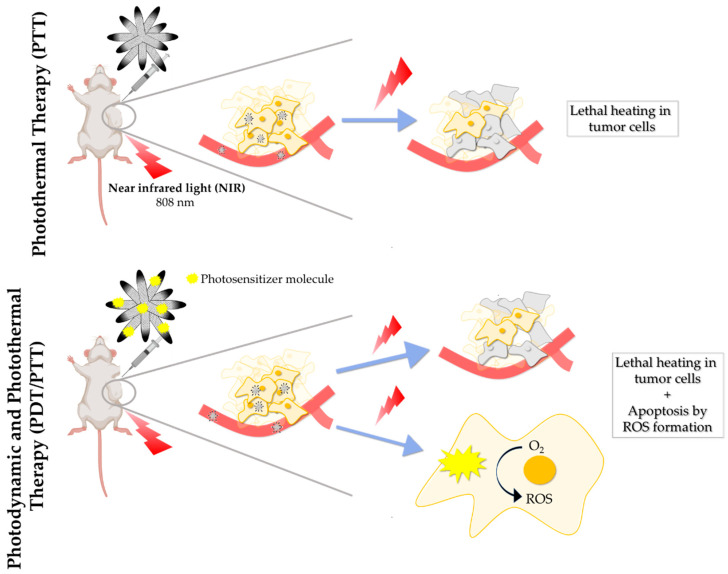
Schematic illustration of PTT and PDT/PTT combination in mice with subcutaneous tumor.

Photothermal therapy (PTT) is a type of phototherapy consisting of an irreversible cell damage induced by heat in a selected tissue area (Figure 6). A photothermic agent acts transforming the absorbed energy by the photons into heat [83]. Considering that temperature is one of the most important parameters when determining cell dynamics and viability, PTT induced hyperthermia (over physiological conditions at 37 °C) can disturb tumor mass growth [97]. For this reason, PTT has become one of the preferential options to design nanotherapies for cancer research in the last decades. Carbon-based nanostructures have emerged as novel photothermic agents for this type of therapy, as they present outstanding absorption properties when irradiated with NIR light [98,99]. SWCNH presents extraordinary photothermal qualities [100]. When excited with NIR light spectrum wavelengths, they produce a localized increase in temperature, which provides lethal heat-shock in tumor tissues, leading to the irreversible damage in cancer cells. SWCNH have already been used as NIR photo absorbers to burn tumor areas using different laser excitation [99], especially at a wavelength of 808 nm [41,100,101].

Apart from their extraordinary properties as photothermal agents, SWCNH have also been employed to design multimodal nanocarriers for molecules that act as photodynamic agents [52]. Photodynamic therapy (PDT) is another type of phototherapy with the aim of achieving cell death by producing ROS in cancer cells. PDT has been applied in clinics since it is considered as a minimally invasive therapeutic strategy. A photosensitizer is administered and, with suitable laser wavelength irradiation in the presence of tissue oxygen, this drives photochemical reactions that lead to ROS production. This causes direct cell death in tumor zones where the laser is irradiating, in addition to localized microvasculature damage [102,103]. SWCNH are currently being considered as delivery systems for potential photosensitizing agents. For example, indocyanine green (ICG) have been investigated as a photodynamic agent to combat 4T1 triple negative breast cancer cells [104]. This compound is an effective light absorber for laser-mediated photodynamics, which is an amphiphilic tricarbocyanine with photosynthetic properties to produce ROS and destroy cancer cells [105]. However, it presents poor stability and degrades rapidly. To solve these inconveniences, Gao et al. have coupled ICG with SWCNH by hydrophobic π–π stacking interactions, obtaining a more stable agent in physiological conditions. Under NIR laser irradiation, ICG led to ROS formation and an increase in temperature until 55.3 °C was achieved in the tumoral tissue irradiated with 808 nm wavelength. This combined photothermal and photodynamic therapy was able to kill triple negative breast cancer cells. In the same study, the nanoaggregate was also tested in vivo in tumor-bearing mice in their hind legs. Efficient inhibition of tumor growth was observed with the combined PDT and PTT [104]. Another study has reported the possibility to perform PDT-PTT combined therapy with SWCNH. Phthalocyanine was proposed as the photosensitizing agent for ROS production attached to SWCNH through hydrophobic π–π stacking interactions [52]. Phthalocyanine is another promising compound with outstanding properties for PDT [106]. SWCNH functionalized with phycocyanin also resulted efficiently for ROS production and PTT in both in vitro (in 4T1 triple negative breast cancer cells) and in vivo (in mice with subcutaneous 4T1 cell tumor formation). Interestingly, the greatest decrease in tumor growth was observed when PDT was combined with laser irradiation. This promoted that SWCNH synergistically acted as photothermal agents together with phycocianin [107]. In conclusion, PDT can be combined with other therapies such as chemotherapy, PTT (Figure 6), or radiotherapy [108]. SWCNH can be considered as remarkable nanocarriers for PDT agents with the advantage to perform synergistic PTT.

### 4.4. SWCNH in Cancer Gene Therapy and Immunosensing

Although the use of SWCNH in cancer therapy has been mainly focused on their performance as carriers for chemotherapeutic and for PTT/PDT agents, other emerging strategies have also been described. One of them is the potential use of these nanoaggregates as nucleic acid delivery systems for a feasible gene therapy. For example, PAMAM dendrimers can be coupled to SWCNH to perform strong electrostatic interactions with DNA and RNA as described above. They improve the nanoparticle solubility and their positive charge attach nucleic acids for transport and delivery [21,83]. One study has reported the association between SWCNH@PAMAM-G4 and a siRNA against cofilin-1, which is an essential protein that regulates the cellular cytoskeleton in prostate cancer cells. Carbon nanoaggregates coupled to dendrimers protected the siRNA from RNAse degradation, being able to carry out an efficient transfection to the PC-3 prostate cancer cells. In addition, the same delivery system has also been described as a carrier for docetaxel chemotherapeutic drug, and its effect was enhanced by the inhibition of cofilin-1 [73].

SWCNH have also been studied for cancer diagnosis as immunosensor nanocarriers. Early detection of malignances is essential in cancer, as it is directly correlated to survival rates. For this reason, it is vital to define cancer biomarkers and adequate detection mechanisms. Immunosensors are based on the specific antigen–antibody interaction. Antibodies against specific biomarkers may be immobilized in different substrates covered with nanoparticles [109]. In this regard, SWCNH have been used as nanocarriers of antibodies against human epididymis protein 4 (HE4), which is an ovarian cancer biomarker [100], and α-fetoprotein, a biomarker associated with liver, ovary, or testicle cancer diagnosis [110]. Therefore, SWCNHs appear as a promising nanoplatform to be combined with other substrates and techniques for antibody binding, detection, and future use of immunosensors.

## 5. Discussion and Conclusions

In the last few decades, cancer has become one of the most harmful diseases, with an increasing prevalence in developed countries. Most of the treatments still have excessive undesired effects. For this reason, rational tumor-specific drug design is essential to avoid toxicity, improve efficacy, and the patient’s life quality overall. SWCNHs have proven their efficacy, easy and simple synthesis process scalable to an industrial scale, and their low toxicity highlighting that they are extraordinary candidates for DDS in cancer. Moreover, they can be simply functionalized to improve their physicochemical characteristics or targeting properties.

Many different carbon-based structures are being developed for the treatment of cancer. Comparative studies are necessary to address which of these materials demonstrate better properties for each specific biomedical application. SWCNH have emerged as interesting carbon-based candidates for chemotherapy and for the rational design of selective DDS. They share the extraordinary properties of graphene nanoparticles, that is, high loading capacities, absorption in the NIR region and straightforward surface chemical functionalization. A specific advantage for SWCNH includes the capacity for high yield mass production at room temperature, without potentially toxic metals. Conical morphology of SWCNH is also interesting for drug entrapment and transport. Oxidation of SWCNH is also an interesting approach for functionalization and attachment of anticancer drugs. For future studies, it is vital to determine the optimal oxidation conditions for the proper incorporation of drugs, as well as to address the number of molecules that can be incorporated.

Further studies are needed to analyze SWCNH biocompatibility to standardize the most adequate functionalization for the design of DDS. Covalent modifications may require prior oxidation. Non-covalent electrostatic or π–π interactions may be also interesting for anticancer drug attachment but sometimes not sufficiently strong. Many ongoing studies are examining the most adequate options and parameters needed for the functionalization of these nanoparticles. Functionalization is essential to understand the toxicity of SWCNH and the physiological excretion pattern of these nanoparticles. Current in vitro and in vivo toxicity studies are not comparable to develop reliable cancer therapies for clinical use. In vitro studies should also include toxicity parameters in non-cancer cell lines since there is little data about toxicity in different healthy cells. In vivo, parameters such as the maximum tolerated dose, stability studies in blood and plasma, and the impact of the administration route should be evaluated. Biocompatibility studies should harmonize the monitorization of parameters such as irritation, edema production, body weight variations in the animal, changes at the tissue level with histological analysis, and the general appearance of the animal welfare. A possible solution might be using standard serum parameters employed in clinics to analyze hepatic and kidney function or inflammatory cytokines after injection of SWCNH. Current studies mainly include histochemical analyzes of SWCNH in different tissues or cells. Verification of their accumulation in mononuclear cells of the liver, lungs, or spleen is also essential, as these cells uptake any strange nano-sized material. Although SWCNH have not shown relevant toxicity, the effects on the immune system need to be further explored.

More studies are needed to understand which is the precise mechanism by which SWCNH can act as direct agents against cancer although mitochondrial inner membrane depolarization has been proposed as a possible molecular mechanism. In contrast, no toxic effects of SWCNH have been observed in healthy umbilical vein endothelial cells, but it is necessary to analyze other types of healthy cells. SWCNH have demonstrated great potential as DDS for chemotherapeutic agents. However, more in vivo evidence is needed to analyze their real efficacy for clinics. SWCNH accumulates mostly in the liver and spleen for approximately one month if they are administered intravenously. Therefore, for organs different to liver and spleen, intratumoral injection or the design of a very specific organ targeting would be recommended. At the cellular level, SWCNH are mainly captured by the macrophages. This means that therapeutic strategies using SWCNH to modulate macrophages and the immune system might be of special interest in the future.

The property of SWCNH to absorb light from NIR has been exploited to increase the temperature on the tumor area. Since other graphene structures also have this property, new studies are required to compare different graphene structures to select the most efficient for thermal therapy in cancer. SWCNH are excellent carriers for photosensitizers. The design of a combined PTT/PDT therapy is feasible and particularly interesting as a potential anticancer therapy. SWCNH have extraordinary potential for PTT and as DDS for PDT due to their specific physicochemical properties and may be further developed for nanotheranostics soon.

The use of SWCNH in targeted gene therapy for cancer has been less studied, but it appears to be promising in this field. Their use as carriers of immunosensors for the early diagnosis of cancer must also be taken into consideration for the design of early cancer diagnosis strategies.

This review has summarized the use of SWCNH in biomedical and pharmacological applications, emphasizing their use as nanotube-derived delivery systems for cancer therapy.

In conclusion, SWCNHs have revealed a myriad of therapeutic and diagnostic opportunities in the field of cancer, which will be further exploited during the next years and may offer new therapeutic alternatives in clinics.

## 6. Future Directions

Future research on SWCNH should focus on the harmonization and standardization of the wide array of existing surface functionalization systems to find the more adequate solution for improving drug loading capacity, targeting properties, photothermal and photodynamic reactivity, and biocompatibility both in vitro and in vivo to selectively treat cancer cells. The attachment of molecules for specific targeting of tumors without affecting healthy tissues or non-cancerous cells is crucial. Covalent and non-covalent functionalization processes will be explored to provide the ideal physicochemical characteristics so that SWCNH become adequate to maximize selectivity in cancer therapy.

## Figures and Tables

**Table 1:** Administration routes of SWCNH.

Administration Route	Dosage	Mice Monitorization	Evaluated Parameters	References
Eye contact	0.02 g/eye	1 h after injection	No irritation response	[55]
Skin contact	0.015 g/site	1 h after injection	No erythema or edema formation	[55]
Oral administration	2000 mg/kg of body weight.	For 14 days	No body weight changes	[55]
Intratracheal instillation	2.25 mg/animal	90-day test period	No clinical symptoms of distress and no changes in a whole-lung microarray analysis	[56]
Intravenous administration	6 mg/kg of body weight.	26 weeks test period	Normal gross appearance and no severe abnormalities in the tissues were found on histological observations	[57]

**Table 2:** Use of SWCNH with chemotherapeutic agents.

Chemotherapy	Functionalization and Union to SWCNH	Target	References
Cisplatin (CDDP)	Incorporation into SWCNH cone	AY-27 rat bladder carcinoma cells	[48]
	Oxidized drug attachment to amine functionalized SWCNH	PC-3 PSMA^+^ prostate cancer cells	[79]
	Drug incorporated with nanoprecipitation [80]	NCI-H460 human lung cancer cells	[78,80,81]
		Balb/c *nu/nu* mice bearing NCI-H460 human lung cancer cells	[78]
Dual cisplatin (CDDP) and doxorubicin (DOX)	Poly(maleic anhydride-alt-1-octadecene) (C18PMH) and methoxypolyethyleneglycol-b-poly-d, l-lactide (mPEG-PLA)	Balb/c mice bearing 4T1 breast cancer cell line	[41]
Doxorubicin (DOX)	Hydrophobic π-π stacking interactions	MCF-7 human breast adenocarcinoma cells	[82]
		4T1 breast cancer cells and Balb/c mice bearing 4T1 cells	[83]
	Polyethylene glycol (PEG)	NCI-H460 human non-small lung cancer cells	[84,85]
		Balb/c *nu/nu* mice bearing NCI-H460 human non-small lung cancer cells	[85]
Methotrexate	1,2-Disteatoyl-sn-glycero-3- phosphoethanolamine–*N*-poly(ethylene glycol)-amine (DSPE–PEG-NH_2_)	Human lung adenocarcinoma (A549) and breast adenocarcinoma (MAD-MB-231) cell line. ICR mice bearing H22 hepatocellular carcinoma cell line.	[86]
Vincristine	Physical adsorption	MCF-7 human breast adenocarcinoma cells and ICR mice bearing H22 hepatocellular carcinoma cell line.	[87]
